# SUMOylation and related post-translational modifications in natural killer cell anti-cancer responses

**DOI:** 10.3389/fcell.2023.1213114

**Published:** 2023-05-25

**Authors:** Rosa Molfetta, Sara Petillo, Marco Cippitelli, Rossella Paolini

**Affiliations:** Department of Molecular Medicine, Laboratory Affiliated to Istituto Pasteur Italia-Fondazione Cenci Bolognetti, Sapienza University of Rome, Rome, Italy

**Keywords:** SUMOylation and related post-translational modifications, NK cell receptors, ligands for NK cell receptors, NK cell immune surveillance, therapeutical strategies

## Abstract

SUMOylation is a reversible modification that involves the covalent attachment of small ubiquitin-like modifier (SUMO) to target proteins, leading to changes in their localization, function, stability, and interactor profile. SUMOylation and additional related post-translational modifications have emerged as important modulators of various biological processes, including regulation of genomic stability and immune responses. Natural killer (NK) cells are innate immune cells that play a critical role in host defense against viral infections and tumors. NK cells can recognize and kill infected or transformed cells without prior sensitization, and their activity is tightly regulated by a balance of activating and inhibitory receptors. Expression of NK cell receptors as well as of their specific ligands on target cells is finely regulated during malignant transformation through the integration of different mechanisms including ubiquitin- and ubiquitin-like post-translational modifications. Our review summarizes the role of SUMOylation and other related pathways in the biology of NK cells with a special emphasis on the regulation of their response against cancer. The development of novel selective inhibitors as useful tools to potentiate NK-cell mediated killing of tumor cells is also briefly discussed.

## 1 Introduction

Natural Killer (NK) cells are cytotoxic innate lymphoid cells involved in the host immune response to viral infections and in cancer immunosurveillance (Chiossone et al., 2018).

The mechanisms of NK cell-mediated killing include the release of lytic granules containing pore-forming proteins and proteases such as perforin and granzymes and the induction of target cell apoptosis by engagement of the death receptors FAS and TNF-related apoptosis-inducing ligand receptor (TRAILR) (Prager and Watzl, 2019).

NK cells also play immunomodulatory functions by producing cytokines including interferon (IFN)γ and chemokines upon engagement of activating receptors and/or in response to stimulatory cytokines (Fauriat et al., 2010; Freeman et al., 2015).

**FIGURE 1:**
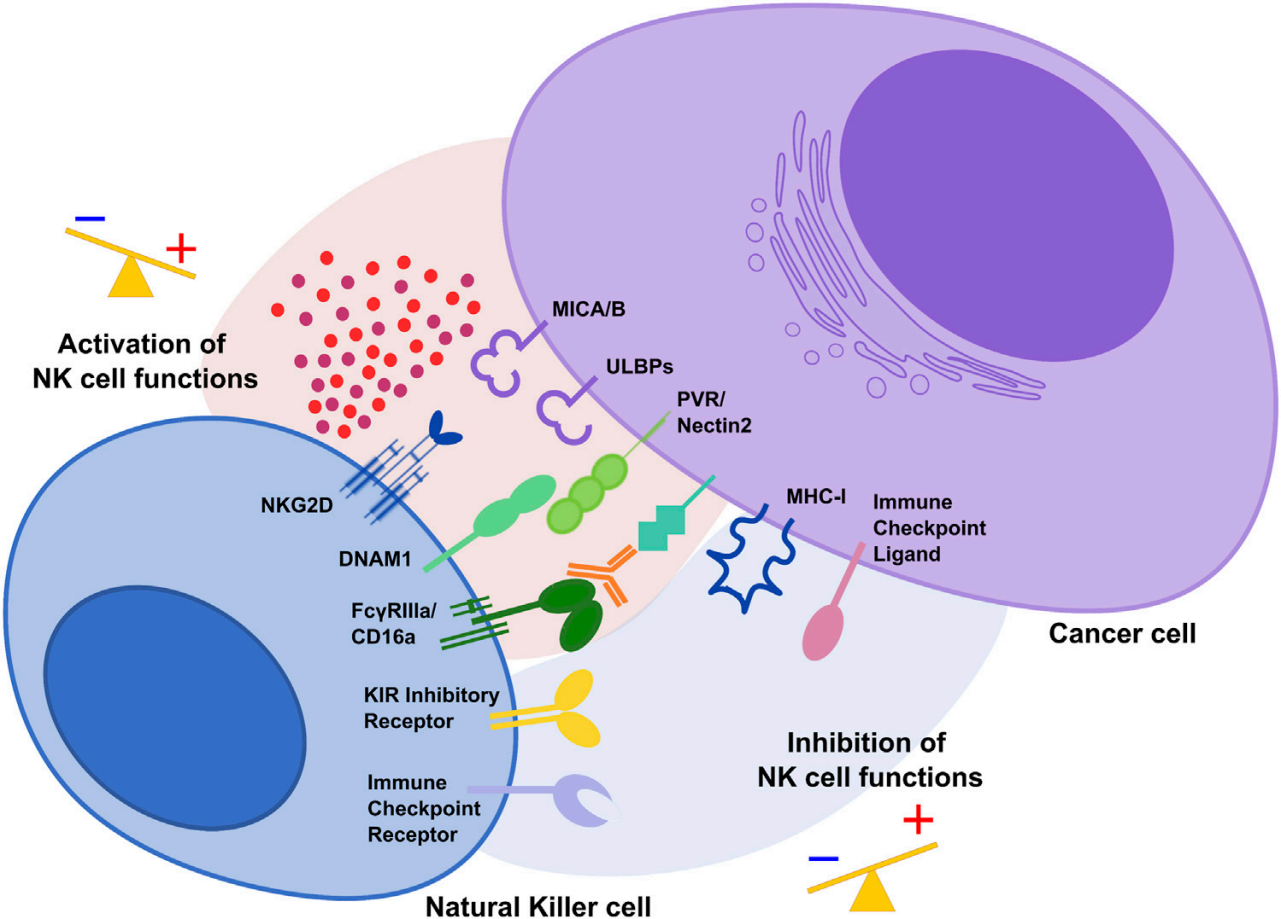
The activating and inhibitory receptor signaling regulates NK cell activation. Cells undergoing stress such as tumor cells lose their MHC class I molecules, ligands for KIR inhibitory receptors on NK cells. At the same time, they acquire stress-associated molecules which act as ligands for the activating receptors NKG2D and DNAM1. Thus, the lack of inhibitory signaling coupled with induction of activating signaling shifts the balance toward NK cell activation, leading to killing of cancer cells. During tumor progression, interaction of immune checkpoint receptors with their cognate ligands diminishes NK cell cytotoxic potential and prevent NK anti-tumor functions.

NK cell activation depends on the integration of tightly regulated signals from inhibitory receptors, including KIR in humans, that recognize “self” Major Histocompatibility Complex (MHC) class I molecules expressed on healthy cells and several activating receptors such as NKG2D and DNAM-1 able to bind stress-induced molecules in infected or transformed cells (Figure 1) (Long et al., 2013; Morvan and Lanier, 2016). During carcinogenesis, tumor cells can modulate the surface expression of the ligands for activating receptors and alter the tumor microenvironment (TME) to evade NK cell-mediated cytotoxicity (Waldhauer and Steinle, 2008; Cerboni et al., 2014; Morvan and Lanier, 2016).

Several mechanisms including post-translational modifications (PTMs) are responsible for the acquisition of a dysfunctional NK cell phenotype characterized by the down-modulation of the main activating receptors and the expression of checkpoint inhibitory receptors able to bind their ligands in the TME to prevent NK cell activation.

In this article, we summarize and discuss the current state of knowledge on SUMOylation and other related PTMs able to regulate NK cell lineage commitment and maturation as well as the expression of activating receptors and their ligands. Finally, we outline current strategies based on the use of PTM inhibitors to potentiate NK-cell immune surveillance against cancer.

## 2 NK cells: from development to antitumor functions

NK cells develop from common lymphoid progenitor cells in the bone marrow (Kondo et al., 1997) and their maturation takes place in the primary and secondary lymphoid organs from where they directly join the circulation (Kim et al., 2002; Scoville et al., 2017).

The progenitors that give rise to NK cells are defined by the differential expression of lineage-specific surface markers. These markers are different between humans and mice, but the regulated expression of critical transcription factors (TFs), such as the T-box transcription factors T-bet and Eomesodermin, controls NK cell-specific development, maturation and functions in both species (Simonetta et al., 2016).

During the development of NK cell receptor repertoire, the interaction of inhibitory receptors with self MHC-I molecules renders NK cells functional and able to distinguish healthy from altered cells that downregulate or fail to express MHC-I molecules, according to the “missing self” hypothesis (Yokoyama and Kim, 2006). NK cells can also recognize upregulated molecules on the surface of transformed cells to efficiently target and kill them.

NK cell mediated cytotoxicity against a variety of spontaneous tumors mainly depends on activating receptors which include the Natural Cytotoxicity Receptors (NCRs), Natural-Killer receptor group 2, member D (NKG2D) and DNAX Accessory Molecule-1 (DNAM1). Indeed, mice deficient in their expression show an increased incidence of tumor development (Gilfillan et al., 2008; Guerra et al., 2008; Iguchi-Manaka et al., 2008; Halfteck et al., 2009; Mentlik James et al., 2013).

NCRs comprise the immunoglobulin-like receptors NKp46 and NKp30, constitutively expressed on all NK cells, and NKp44 expressed only on IL-2 activated NK cells (Pazina et al., 2017).

NKp46 engagement induces NK cell cytotoxicity and cytokine release upon interaction with viral components such as hemagglutinin, the soluble complement factor P as well as heparan sulfate proteoglycan, which is expressed by different tumors (Sivori et al., 1997; Narni-Mancinelli et al., 2017).

NKp44 ligands include Proliferating Cell Nuclear Antigen (PCNA), platelet-derived growth factor DD (PDGF-DD), nidogen-1, and NKp44L, an isomer of mixed-lineage leukemia-5 protein (MLL5). All of them by binding NKp44 can improve tumor sensitivity to NK cell cytotoxicity (Baychelier et al., 2013). However, when PCNA is aberrantly expressed on the surface of tumor cells it can associate with HLA-I molecules forming an inhibitory ligand complex (Rosental et al., 2011).

NKp30 ligands include B7-H6 and the HLA-B-associated transcript 3 protein (BAT3), also known as BAG6, both able to promote NK cell cytotoxicity against tumor cells and soluble galectin-3 implicated in NK cell tumor evasion (von Strandmann et al., 2007; Brandt et al., 2009; Wang et al., 2014).

Additionally, all NCRs can recognize heparan sulfate glycosaminoglycans (HS-GAGs) which are significantly upregulated in tumor cells.

NKG2D is expressed on NK cells but also on CD8^+^αβ T cells, γδ T cells, and activated CD4^+^αβ T cells (Ullrich et al., 2013; Marcus et al., 2014; Lanier, 2015). To signal, NKG2D associates with DNAX-activating protein of 10 kDa (DAP10) which has a YINM motif that induces PI3 kinase and Grb2-Vav signaling. However, in murine NK cells NKG2D can also associate with DAP12, containing an immune tyrosine-based activation motif (Diefenbach et al., 2002; Gilfillan et al., 2002).

Human NKG2D ligands (NKG2DLs) consist of two families of polymorphic molecules structurally related to MHC class I: MHC class I related chains (MIC)A and B, mainly expressed as transmembrane proteins, and six UL16 binding proteins (ULBPs) that can be associated with the membrane via a transmembrane domain or by GPI anchor (Raulet et al., 2013; Lanier, 2015; Zingoni et al., 2018). Murine NKG2DLs include members of the Rae-1 family, which are orthologs of human ULPBs, murine UL16-binding protein-like transcript 1 (MULT1) and the H60a-c family (Raulet et al., 2013).

DNAM1 (CD226) belongs to the immunoglobulin receptor family and is expressed on the surface of NK cells, T cells, monocytes and subsets of B cells (Shibuya et al., 1996; de Andrade et al., 2014). It can transmit activating signals through the association with lymphocyte function-associated antigen 1 (LFA-1) upon binding with its ligands Nectin2 (CD112) and PVR (CD155) (Bottino et al., 2003; Tahara-Hanaoka et al., 2004; Chan et al., 2014a). In addition to DNAM1, PVR can also interact with CD96 (TACTILE) and with TIGIT, checkpoint receptors that can counterbalance the DNAM1 mediated activating signals (Chan et al., 2014b; Molfetta et al., 2020).

Under normal conditions, most of the above-mentioned NK cell activating ligands are absent in autologous cells but their expression is induced upon transformation on a broad panel of tumors (Groh et al., 1999; Pende et al., 2001; Friese et al., 2003; Jinushi et al., 2003; Salih et al., 2003; Textor et al., 2016). On the other hand, healthy cells express low levels of Nectin2 and PVR but their amount increases on malignant cells promoting DNAM1-dependent NK cell cytotoxicity (Pende et al., 2005; Carlsten et al., 2007; El-Sherbiny et al., 2007; Lakshmikanth et al., 2009).

Thus, NK cell ability to distinguish their targets is dictated by a tight regulation of activating ligands on the surface of transformed cells.

Of note, the low affinity receptor for immunoglobulin G (FcγRIIIA, CD16) also contributes to tumor clearance, as revealed by the augment of antibody-dependent cell-mediated cytotoxicity (ADCC) by NK cells upon the use of therapeutic IgG monoclonal antibodies (Mentlik James et al., 2013; Battella et al., 2016).

## 3 Ubiquitin and ubiquitin-like modifications able to regulate NK cell functions

Ubiquitination, SUMOylation and Neddylation are PTMs whereby small highly conserved proteins called ubiquitin (Ub), SUMO (Small Ubiquitin-related MOdifier) and NEDD8 (Neural Precursor Cell Expressed, Developmentally Downregulated 8), respectively, are covalently bound to lysine (K) residues of target proteins through the sequential action of selective E1, E2, and E3 enzymes that are frequently upregulated during malignant transformation (Chen et al., 2020; Xue et al., 2020).

**FIGURE 2:**
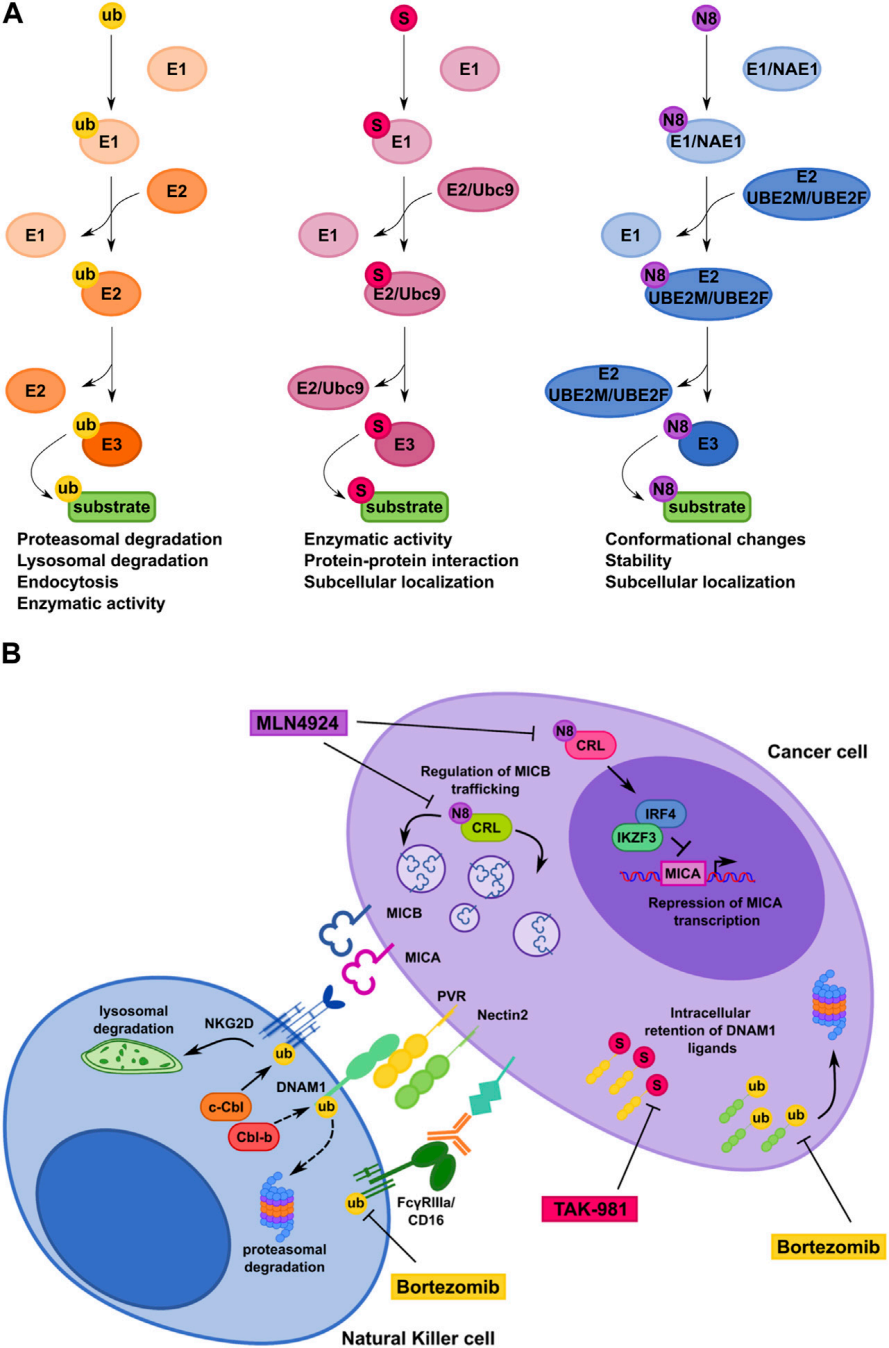
Comparison of ubiquitin, SUMO and NEDD8 conjugation pathways controlling NK cell-mediated recognition of tumor cells. **(A)** Schematic representation of the ubiquitin pathway and the ubiquitin-like protein modifications SUMOylation and Neddylation. Ub, ubiquitin; S, SUMO; N8, NEDD8. **(B)** Model depicting how ubiquitin, SUMO and NEDD8 pathways regulate CD16, NKG2D and DNAM-1 surface expression on NK cells (left) and NKG2D and DNAM-1 ligand expression on cancer cell (right). Therapeutic interventional strategies aimed to prevent post-translational mechanisms affecting expression of NK cell receptors and their cognate ligands on cancer cells are also shown.

Despite substantial mechanistic similarities between Ub- and Ub-like modifications, specific properties of each system can determine the fate of the modified protein (Figure 2A).

Ubiquitination through the covalent addition of a single Ub molecule or various Ub chains regulate numerous biological and functional events. For instance, K48 poly-Ub chain formation is associated with protein degradation by the 26S proteasome, whereas the addition of a single Ub molecule through K63 promote non-degradative fates including membrane protein endocytosis (Passmore and Barford, 2004; Sadowski et al., 2012).

De-ubiquitinating enzymes (DUBs) remove Ub chains from proteins to maintain intracellular Ub levels, and the interplay between ubiquitinating and de-ubiquitinating enzymes is necessary to maintain cellular homeostasis (Zou and Lin, 2021).

SUMOylation targets a consensus motif, ΨKXE/D (where *Ψ* represents a large hydrophobic residue and X any amino acid), in which the lysine residue serves as acceptor site of different SUMO family members. The reaction is catalyzed by a dimeric E1 conjugating enzyme (SAE1/2) and a unique E2 enzyme called Ubc9 (ubiquitin conjugating enzyme 9), which works together with a dozen of E3 SUMO ligases to ensure substrate specificity (Flotho and Melchior, 2013).

SUMO is mainly conjugated as a monomer, however some SUMO members share with Ub the ability to form SUMO chains. SUMOylated substrates can recruit proteins bearing SUMO interaction motifs (SIMs) and undergo conformational changes that affect their stability, subcellular localization and functions (Flotho and Melchior, 2013).

Although SUMOylation of target proteins in general does not lead to their proteasomal degradation, SUMO and Ub pathways often act sequentially (Lallemand-Breitenbach et al., 2008; Tatham et al., 2008) and/or can synergize to induce substrate degradation, as demonstrated for IκBα (Aillet et al., 2012).

As for ubiquitination, SUMOylation can be reverted by de-SUMOylated enzymes including the family of Sentrin/SUMO-specific proteases (SENP1-3 and SENP5-7) (Tokarz and Woźniak, 2021). SUMOylation and de-SUMOylation have been shown to control wide arrays of cellular activities including cell cycle, DNA repair, transcription and chromosome remodeling (Celen and Sahin, 2020).

Analogous to ubiquitination and SUMOylation, Neddylation also uses E1 and E2 enzymes as well as multiple E3 ligases to ensure substrate modification (Kamitani et al., 1997). It is well recognized that the main substrates for Neddylation are cullins, a family of multi-components Cullin-RING ubiquitin Ligases (CRLs): Cullin Neddylation leads to a conformational change, which allows the simultaneous binding of other components to cullins and hence the assembly of a functional Ub ligase big complex (Liu et al., 2002; Ohh et al., 2002; Sakata et al., 2007). Notably, a dynamic cycling of cullin Neddylation and de-Neddylation is required for the optimal CRL activity since perturbations of either Neddylation or de-Neddylation cause accumulation of CRL specific substrates.

Besides cullins, a growing number of non-cullin targets has also been reported in a process referred as non-canonical Neddylation (Enchev et al., 2015). This process mainly affects protein intracellular localization and functions as well as protein stability (Brillantes and Beaulieu, 2019; Zou and Zhang, 2021).

### 3.1 Post-translational modifications and regulation of NK cell maturation

Development and maturation of NK cells is regulated by progressive and coordinated TF activity (Brillantes and Beaulieu, 2019; Bi and Wang, 2020). In this regard, TFs such as E4BP4, TOX, ETS1, and ID2 are required for NK cell lineage commitment, while others such as ID2, T-bet, Eomes and ZEB2 for NK cell maturation, where they promote the expression of genes coding for effector molecules, receptors responsible for egress, and cell-surface maturation markers (Brillantes and Beaulieu, 2019; Bi and Wang, 2020).

In this scenario, SUMOylation and ubiquitination have been described to regulate the activity of these TFs in cancer models and during lymphocyte differentiation and activation.

E4BP4 is a basic leucine zipper TF able to regulate different biological pathways, ranging from circadian rhythms to lymphocyte differentiation and function (Cowell, 2002; Yin et al., 2017). E4BP4 is expressed by CLPs, before other NK lineage-defining TFs, and its expression increases as NK cells undergo differentiation and maturation (Male et al., 2014).

Studies in E4BP4^−/−^ mice showed that the activity of E4BP4 is crucial for the generation of ILC subsets and NK cells, although it is dispensable for the development of “tissue-resident” NK cell populations in the salivary gland and liver (Gascoyne et al., 2009; Kamizono et al., 2009; Cortez et al., 2014; Crotta et al., 2014; Seillet et al., 2014; Yu et al., 2014).

Different stimuli can regulate the expression of E4BP4 such as IL-7 in ILC progenitors and IL-15 in NK cells (Geiger et al., 2014; Male et al., 2014; Marçais et al., 2014; Yang et al., 2015). Importantly, the activity of this TF has been shown to be strongly regulated by SUMOylation. In this context, mutations of the SUMOylation sites in the E4BP4, lead to increased transcriptional activity and higher production of NK cells when compared to the WT protein. These observations indicate that E4BP4 is critical for early NK cell development and function, and that control of E4BP4 activity by PTM such as SUMOylation can have implications for the production and development of immunotherapeutic strategies using NK cells.

In a different PTM pathway, ubiquitination and deubiquitination reactions has been well characterized as critical for NK cell maturation and anti-cancer activity. In particular, the activity of the protein MYSM1 (Myb-like, SWIRM, and MPN domains-containing protein 1), a histone H2A DUB reported to induce H2A deubiquitination and activation of several target genes in cancer, has been shown to regulate NK cell maturation downstream of IL-15 signaling, with no activity on NK lineage specification and early development (Nandakumar et al., 2013).

In this context, also the DUB Otub1 has been shown to control the maturation and activation of NK cells (Zhou et al., 2019). Deletion of Otub1 in mice had no effect on total NK cell numbers in the spleen, but it significantly increased the frequency of stage 4 mature NK cells. Importantly, Otub1-KO NK cells were more responsive to cytokine-stimulated activation with increased granzyme B and CCL5 expression. Mechanistically, Otub1 is a critical modulator of the IL-15-activated ubiquitination of the AKT kinase, important for metabolic reprogramming, activation, and homeostatic lymphocyte maintenance. Interestingly, deletion of Otub1 in mice was associated with tumor rejection with increased infiltration of NK cells, thus functioning as a checkpoint for IL-15 signaling (Zhou et al., 2019).

### 3.2 PTMs regulate the expression of NK cell-activating receptors and of their cognate ligands on tumor cells

NK cells represent the first line of defense against cancer, and tumor progression is usually accompanied by a decline of their functions. Activating receptors are frequently down-modulated and rendered functionally inactive on NK cells derived by patients affected by different kind of tumors (Costello et al., 2002; Groh et al., 2002; Doubrovina et al., 2003; Oppenheim et al., 2005; Fauriat et al., 2007; Coudert et al., 2008; Carlsten et al., 2009; Garcia-Iglesias et al., 2009; Carlsten et al., 2010; Sanchez-Correa et al., 2012).

Even though the mechanisms underlying NCRs down-modulation are still undefined, the decreased surface expression of engaged CD16, NKG2D, and DNAM1 mainly occurs through Ub-dependent endocytosis (Paolini et al., 1999; Molfetta et al., 2014a; Quatrini et al., 2015; Molfetta et al., 2016; Braun et al., 2020).

NKG2D engagement is rapidly followed by DAP10 ubiquitination required for receptor internalization and subsequent lysosomal degradation (Molfetta et al., 2014a; Quatrini et al., 2015; Molfetta et al., 2016). Of note, NKG2D downregulation also cross-tolerizes other NK cell activating receptors (Oppenheim et al., 2005; Coudert et al., 2008; Hanaoka et al., 2010; Koch et al., 2017; Milito et al., 2023).

Ligand-dependent DNAM1 internalization on NK cells likely involves Ub modification, as formally demonstrated in murine CD8^+^ T cells (Braun et al., 2020).

On human NK cells, CD16 aggregation in response to antibody-coated tumor cells is followed by ubiquitin-dependent CD16ζ subunit endocytosis and lysosomal degradation (Paolini et al., 1999; Molfetta et al., 2014b). Similarly, anti-CD20 opsonized tumor cells promote CD16 clearance from NK cell surface followed by a dramatic reduction of ADCC (Capuano et al., 2015; Capuano et al., 2017).

Altogether these results demonstrate that interaction with ligand-expressing tumor cells and/or monoclonal antibody-based therapies downregulate activating receptor with a mechanism that involves the Ub pathway.

Whether other PTMs, including SUMOylation, impact on activating NK cell receptor expression remains unexplored.

During malignant transformation different stressful stimuli are responsible for NK cell activating ligand regulation on tumor cells. The implication of transcriptional mechanisms is quite well known and deeply described (Raulet et al., 2013; Cerboni et al., 2014) and recently a role for PTMs has also been envisaged (Molfetta et al., 2019a).

In this regard, several data demonstrate that surface expression of human NKG2DLs is regulated by post-translational mechanisms (Fuertes et al., 2008; Aguera-Gonzalez et al., 2009; Fernandez-Messina et al., 2016; Soriani et al., 2016; Bilotta et al., 2019). In melanoma cells, MICA is retained in the endoplasmic reticulum and degraded by proteasome (Fuertes et al., 2008), while in different tumor cell lines MICB and ULBP1 are both continuously internalized and targeted to lysosomal or proteasomal degradation, respectively (Aguera-Gonzalez et al., 2009; Fernandez-Messina et al., 2016; Soriani et al., 2016; Bilotta et al., 2019).

Regarding DNAM1Ls, activation of Unfolded Protein Response promotes internalization and degradation of PVR in hepatocellular carcinoma (Gong et al., 2014). Moreover, in MM cells SUMO and Ub pathways regulate PVR and Nectin2 surface expression, respectively (Zitti et al., 2017; Molfetta et al., 2019b). In particular, our group demonstrated that PVR is SUMOylated and prevalently expressed as intracellular pool in several MM cell lines. Accordingly, inhibition of the SUMO pathway promotes PVR translocation to the cell surface rendering MM cells more susceptible to DNAM1-mediated NK cell cytotoxicity (Zitti et al., 2017). Notably, the SUMO pathway regulates PVR surface expression in tumors other than MM, supporting a more general role for SUMOylation in regulating tumor cell recognition and killing by NK cells (Zitti et al., 2017). Whether the SUMO pathway regulates the expression of NK cell activating ligands different than PVR is currently unknown.

Thus, SUMOylation and other related PTMs represent novel potential targets for therapeutic intervention aimed to improve NK cell-mediated tumor surveillance by promoting activating ligand expression in transformed cells (Figure 2B).

## 4 Targeting post-translational mechanisms to regulate NK cell-mediated recognition and killing of cancer cells

The specific enzymes involved in human NK cell activating ligand modification by the Ub/proteasome pathway have not been identified yet. However, it is possible to change the fate of ubiquitinated ligands by the use of proteasome inhibitors (Morrow et al., 2015).

Bortezomib, Carfilzomib and Ixazomib are three FDA-approved proteasome inhibitors already used as chemotherapeutic drugs for relapsed and/or refractory MM patients and for the treatment of other hematological malignancies (Morrow et al., 2015; Schlafer et al., 2017). Notably, low doses of bortezomib increase the expression of NKG2D and DNAM-1Ls on MM cells and result in enhanced NK-cell susceptibility (Soriani et al., 2009; Niu et al., 2017; Fionda et al., 2018).

Regarding SUMO pathway, Ginkgolic acid was the first compound identified to inhibit the formation of E1-SUMO intermediates (Fukuda et al., 2009). Notably, Ginkgolic acid increase PVR expression on MM cells rendering them more susceptible to NK cell mediated-lysis (Zitti et al., 2017). However, inhibition of the SUMO pathway occurs at micromolar range and can have several non-SUMO-related effects. Recent advances in the development of synthetic E1-SUMO inhibitors including ginkgolic acid derivatives and, most recently, TAK-981 have enabled more specific and efficient targeting of the SUMO pathway (Kumar et al., 2016; Brackett et al., 2020; Langston et al., 2021). TAK-981 is currently involved in phase 1 clinical trials for the treatment of patients with solid tumors and lymphomas (Lightcap et al., 2021). Of note, TAK-981 treatment promotes anti-tumor innate immune responses through activation of type I interferon (IFN-I) signaling that enhances *ex-vivo* macrophage phagocytosis and NK cell cytotoxicity (Nakamura et al., 2022). However, the mechanism by which TAK-981 activates IFN-I responses is still unclear and need further investigation.

MLN4924 (Pevonedistat) is a first-in-class inhibitor of NEDD8-activating enzyme currently involved in phase I/II/III clinical trials for patients suffering from solid and hematological malignancies (Nawrocki et al., 2012; Sarantopoulos et al., 2016; Shah et al., 2016). Notably, MLN4924 increases the expression of NKG2DLs on MM cells, making these cells more susceptible to NK cell degranulation and killing (Petillo et al., 2021). In particular, MICA expression is regulated at mRNA level as result of an increased promoter activity after the inhibition of the transcriptional repressors IRF4 and IKZF3. Differently, MLN4924 induces accumulation of MICB on the plasma membrane with no change of its mRNA levels, indicating a post-translational regulatory mechanism (Petillo et al., 2021).

## 5 Discussion

In conclusion, different PTMs regulate NK cell-mediated surveillance against tumors.

The Ub pathway contributes to downregulate the surface expression of activating NK cell receptors engaged by their respective ligands.

On tumor cells, several NK cell activating ligands are retained intracellularly and/or degraded upon ubiquitination or SUMOylation. Moreover, inhibition of Neddylation upregulates cell surface expression of NKG2DLs on MM cells, making them more efficient to activate NK cell degranulation.

Intriguingly, several inhibitors of those PTMs have been already developed and their use in combination with conventional therapies represents a useful tool to potentiate NK-cell mediated recognition and killing of tumor cells by preserving activating ligand expression on their surface.

Given the reversible nature of SUMOylation and the other related PTMs, it is important to deeply consider the balance between all the potential regulators of those pathways to make their pharmacological inhibition an attractive therapeutic strategy for the treatment of cancer patients.

Moreover, in the future, appropriate preclinical models including an intact tumor microenvironment are needed to explore the full therapeutic potential of SUMO and SUMO related pathway inhibitors on NK cell-mediated anti-cancer responses.
